# Respiratory Exercises in the Treatment of Disease

**Published:** 1898-12

**Authors:** 


**Respiratory Exercises in the Treatment of Disease.** By Harry Campbell, M.D. Pp. viii., 200. London: Baillière, Tindall and Cox. 1898.

Dr. Campbell has come to the conclusion that properly devised respiratory exercises have a great therapeutic value, and his present work is the outgrowth of a larger one on the mechanical treatment of heart disease, in which he has been in a measure forestalled by the physicians of Nauheim. The mode of treatment being founded on physiological principles, it seemed necessary to define these principles in a series of somewhat technical chapters extending over nearly 150 pages. The following six chapters are the practical outcome of the previous theoretical considerations and their application. The number and variety of the exercises suggested almost rival the elaborate series of passive exercises adopted by the advocates of Nauheim treatment; indeed, these movements seem to replace those of the limbs in stimulating the circulation through heart and lungs. Both active and passive breathing exercises may be used, alone or conjoined with other active exercises. "The chief drawback to the treatment . . . . is getting the patient to persevere in it long enough. It involves a considerable sacrifice of time, and is apt to grow irksome. Few good results in this world, however, can be obtained without both pains and patience, and certainly the end gained in this instance is worthy the cost." We wish the author all success in his attempt to train and develop the possibilities of respiratory exercise in the treatment of pulmonary, cardiac, nervous, and other apparatus.

